# Chromatographic Study of Novel Heteronuclear Complexes with Schiff Base as Main Ligand

**DOI:** 10.1007/s10337-014-2664-2

**Published:** 2014-03-26

**Authors:** Agnieszka Wronka, Irena Malinowska, Wiesława Ferenc, Beata Cristovao

**Affiliations:** 1 Department of Planar Chromatography, Chair of Physical Chemistry, Faculty of Chemistry, Maria Curie-Skłodowska University, Plac Marii Curie-Skłodowskiej 3, 216 20-03 Lublin, Poland; 2 Department of General and Coordination Chemistry, Maria Curie-Skłodowska University, Lublin, Poland

**Keywords:** High-performance thin layer chromatography, Magnetochromatography, Electrochromatography heteronuclear, Lipophilicity, Heteronuclear Schiff base complexes

## Abstract

The properties of 12 new heterodi- and heterotrinuclear complexes having general formulae [Cu_2_Ln(L)_2_(NO_3_)(H_2_O)_2_](NO_3_)_2_·3H_2_O [where Ln = Pr (1), Nd (2), Sm (3) and Eu (4)], and [CuLn(L)(NO_3_)_2_(H_2_O)_3_MeOH]NO_3_·MeOH [where Ln = Gd (5), Tb (6), Dy (7), Ho (8), Ef (9), Tm (10), Yb (11) and Lu (12)], and their main ligand [L = C_19_H_18_N_2_O_4_Br_2_ = *N*,*N’*-bis(5-bromo-3-methoxysalicylidene)propylene-1,3-diamine] have been characterized by chromatographic analyses. The parameter of relative lipophilicity (*R*_M0_) of the tested compounds was determined experimentally by reversed-phase high-performance thin layer chromatography method with mixtures of methanol and water as a mobile phase. We also described interactions between chromatographed substances and various surfaces (silica—SiO_2_ and modified by hydrocarbon chains—RP-2, RP-8, RP-18 phases). This study also investigates the effect of pH of the mobile phase on the retention on the polar stationary phase. Thin layer chromatography combined with magnetic and electric field has been proposed as a complementary method for the determination of physicochemical properties of the investigated compounds. The chromatograms in the field and outside of it were developed simultaneously in three identical chromatographic chambers. One of them was placed in external magnetic field of 0.4 T inductivity, and the second in external electrical field. In magnetic and electric fields, retention of some complexes changed, which indicated that the presence of these fields influenced physicochemical properties of the compounds and their interactions with the stationary phase.

## Introduction

The chemistry of coordination compounds is an important and challenging area of modern chemistry. Significant research progress in this discipline has been observed in last few years focusing on physical properties of coordination compounds such as solubility, structural and magnetic features. Complex compounds have been investigated using various methods, such as IR, Raman spectroscopy or X-ray analysis. Those methods give us some information about the properties of the complexes in solid state, but their characteristics in solutes have been still a very interesting problem. Especially that there are no valid procedures for there types of research. That is why liquid chromatography has been proposed as a method of choice.

The investigations of physicochemical properties of coordination compounds, not only as solids, but also in liquid state seem to be very important. The activities of these complexes being changed depending on the environments around the central ion may create various possibilities of their practical applications resulting from the potential sites of their biological action. A search in the literature revealed that the crystal structures of Schiff bases heteronuclear compounds have been well developed [1], but there is little known about their physicochemical properties and behavior in the solutions.

Therefore, we decided to undertake the preliminary study of their behaviour also in the chromatographic aspects in the water and methanol solutions to know the influence of those types of solutes on the activity of the 3*d*–4*f* heteronuclear magnetic centers and to consider the mechanism of their effects under such conditions.

In recent years, there has been a growing interest in the synthesis, structure and physicochemical properties of heteronuclear compounds containing simultaneously 3*d* and 4*f* metal centers due to the variety of their potential applications mainly in bioinorganic chemistry [2–6], magnetochemistry [7, 8], separation processes, luminescence, environmental chemistry, electrochemistry and catalysis [9].

These kinds of compounds belong to molecular magnets being used for the generation of energy and its storage and for the production of charge-transfer superconductors to be characterized by high electrical conductivity at low temperature. As “building blocks”, the high-spin molecular groups have interesting magnetic properties being used in quick electrical switches. They have also application for the production of the devices for the retention of numeral information with the great recording density. Molecular magnets slightly soluble in water can reduce the level of pesticides in food or water [10]. Magnetism of molecular complexes simultaneously containing *d* and *f* transition metal ions has been the subject of investigation in the last few years. According to works of Pasatoiu [11] or Benelli [12], the influence of magnetic field on powder samples of heteronuclear *d* and *f* electron metal complexes was observed. The complexes have different magnetic properties (dia-, para- or ferromagnetic). For that reason the observation of our investigated complex behaviours in solution in magnetic field seems to be an interesting research problem. For that purpose, the technique known in literature as magnetochromatography [13–17], may be used.

The reason for the characterization of transition metals complexes containing Schiff bases lies also in their biological and catalytic activity in many reactions [2–6]. They show also the antimicrobial activity [18]. In the presented research, 12 newly synthesized heteronuclear Schiff base complexes were investigated for lipophilic properties using liquid chromatography. Lipophilicity being a molecular property of solute–solvent interactions, characterized generally in a term of partition coefficients between polar and apolar solvent system.

Concluding, in presented work, thin layer chromatography being combined with magnetic and electric fields has been proposed as complementary method for the determination of physicochemical properties of investigated coordination compounds. The retention analysis of those complexes may give us some information about their affinity to different stationary phases, behaviour in various solutions and about the influence of the central ion on the proposed systems.

## Experimental

### Reagents and Materials

**Fig. 1:**
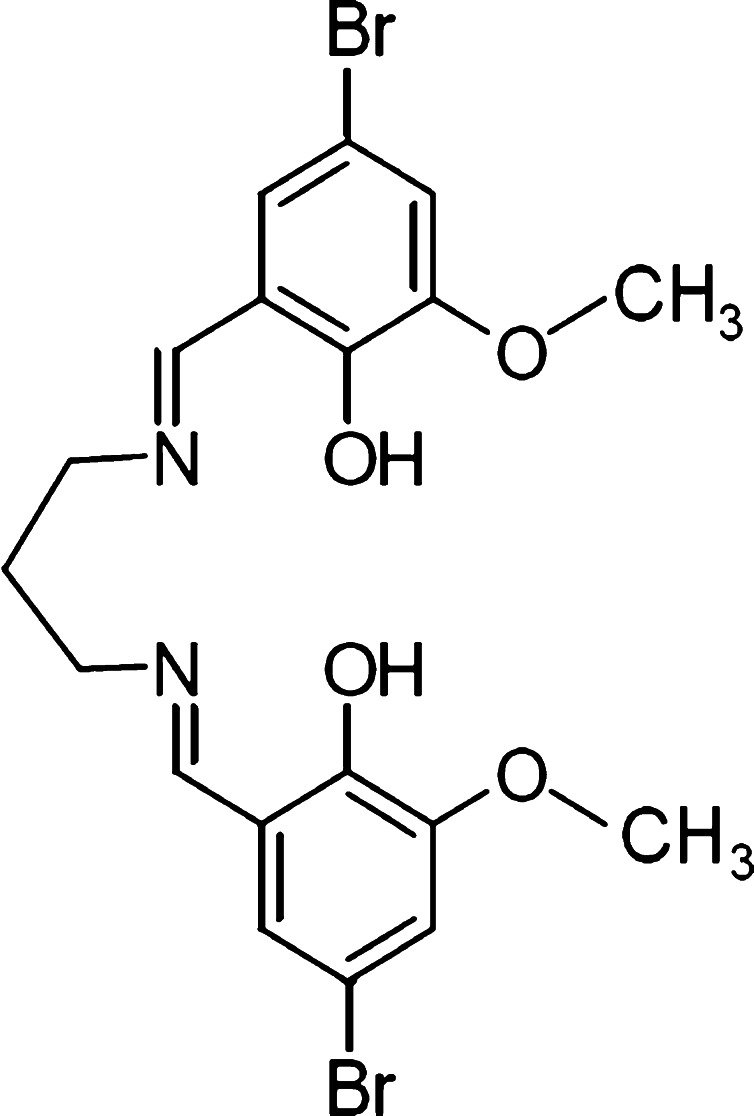
The chemical structure of ligand molecule [23]

**Fig. 2:**
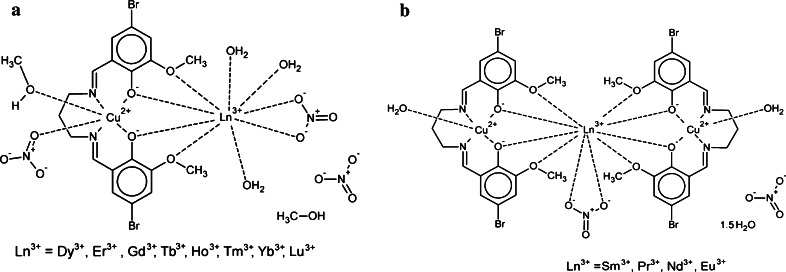
Schematic diagram of di- and trinuclear complexes in solid state [23]

All chemicals and solvents used in our investigations were of commercially available reagent grade and were used without further purification. Methanol, di-sodium hydrogen phosphate dehydrate and citric acid were supplied by Merck (Darmstadt, Germany). Ionic liquid: 1-butyl-3-methylimidazolium tetrafluoroborate was obtained from Sigma-Aldrich. The citrate buffers were prepared from 0.02 M Na_2_HPO_4_ and 0.01 M citric acid mixed together in appropriate proportion. Before use, the buffers were vacuum filtrated through a 0.45-µm membrane filter. The pH value of the buffer was measured before the addition of organic modifier. Distilled water was obtained from Direct-Q UV apparatus (Millipore). All investigated compounds were synthesized at the Department of General and Coordination Chemistry, and their structures were determined in Crystallographic Department, Maria Curie-Skłodowska University in Lublin (Poland) (Figs. 1, 2) [9, 19–22].

### Chromatographic Measurements

All chromatographic measurements were carried out in unsaturated sandwich chambers.

#### High-Performance Thin Layer Chromatography (HPTLC)

Our experiment was performed on commercially HPTLC NP and RP plates. In primary experiment, HPTLC chromatography was carried on 2 × 10 cm RP-18 WF_254s_ HPTLC plates (for ligand) and 10 × 10 cm RP-2 WF_254s_ HPTLC plates (for all complexes) from Merck (Darmstadt). For the determination of the lipophilicity, the mobile phase consisting methanol and water content between 0.5 and 1 organic solvent concentration (for ligand) and between 0.7 and 0.1 organic solvent concentration (for complexes), expressed as volume fraction v/v, in constant steps of 0.05, was used.

The investigated compounds were dissolved in methanol (1 g/L), and 1 μL solutions were separately spotted onto plates. All measurements were carried out at ambient temperature. The chromatograms were developed and plates were dried. The spots were observed under a UV lamp (254 nm). The *R*_F_ values were obtained from three independent measurements. *R*_M_ values of tested compounds were calculated from the experimental *R*_F_ values by use of the Eq. (1):

1 $$ R_{\text{M}} = { \log }\left( {\frac{{ 1 { - }R_{\text{F}} }}{{R_{\text{F}} }}} \right) $$

#### Magnetochromatography

The measurements of magnetic field influence were carried out using TLC technique. For that purpose, special modification of chromatographic instruments was performed. “Sandwich” type of chromatographic chamber was placed between a pair of neodymium magnets (after dismounting all of its ferro- and paramagnetic parts). In our experiments, 0.44-T inductivity was used. Chromatograms were developed in chambers with external, perpendicular to the plane magnetic field and without it simultaneously. The following mobile phases were applied:

1. methanol: citric buffer pH = 3 [9:1 (v/v)];
2. methanol: citric buffer pH = 8 [9:1 (v/v)];
3. methanol: water [9:1 (v/v)].

#### Electrochromatography

Plates were developed in a modified horizontal Chromdes (Poland) sandwich chambers placed in the experimental box with electric field (*E* = 1.5 kV/cm) and without electric field simultaneously. The direction of the electric field was parallel to the surface of stationary phase layer and to the direction of the mobile phase. Voltage has been applied by Vera Labeco high voltage feeder, by placing platinum electrodes situated inside chromatographic chamber. The experimental box was closed and after that high voltage was applied [24–26]. In all our experiments, the same solvent mixtures were used on both sides. Novel Schiff base derivatives were analyzed with the same mobile phase as in the magnetochromatography.

## Results and Discussion

The retention data of presented novel Schiff base derivatives have not been described in the literature before, and now they are presented for the first time in this paper.

### Interaction with Different Stationary Phases

In order to determine how the investigated compounds interact with some kind of surfaces, the various HPTLC layers and different solvent systems were performed. The first results were obtained on silica gel plates. Retention of all investigated solutes (complexes and their ligand) was very strong (for example in methanol, *R*_F_ value is smaller than 0.2), therefore investigations in this system have no analytical sense. So, strong interactions between ligand and silica gel, based as a stationary phase, prove its strong affinity with surfaces hydroxyl groups caused by Schiff bases. In the case of complex compounds, as it was determined in previous papers, the coordination bonds are being formed between the central ions and basic ligand centre. Strong retention of this kind of compounds may be caused by:

1. Interaction of the base part of ligand with polar surface, which means that in investigated system covalent bonds are destroyed;
2. Interaction of central ion with silica surface.

In RP systems with RP-18 and RP-8 stationary phases, in a similar way to NP systems, retention at all investigated complexes was very strong.

It is very unusual that retention of compounds is very strong under both NP and RP systems. We supposed that analytes can interact with residual silanol groups of silica matrix. Coverage of the silanol groups in the chemically bonded phases is less than 50–60 %, therefore deleterious effects of free silanols are not fully removed [27, 28]. Silanol interaction may be reduced, e.g., by the addition of ionic liquid, which interacts more strongly with residual silanols, allowing the less basic compound to interact solely with the alkyl ligand of the stationary phase [29]. We found that ionic liquids of the imidazolium tetrafluoroborate class (1-butyl-3-methylimidazolium tetrafluoroborate), added to mobile phase at concentration 1 % (v/v), blocked free silanol groups and retention of all investigated compounds decreases. In chromatographic systems without ionic liquid all investigated solutes practically stayed at the start line in RP systems, even in pure methanol as mobile phase. The strong proton-acceptor properties of ionic liquids could be of use to suppress the known deleterious effect of free silanols on liquid chromatographic separation of our compounds. Ligand retention values in RP-18 systems with methanol: water as a mobile phase remains within an analytical range (between *R*_F_ values 0.2–0.8), within a wide range of organic modifier quantity. Coordination compound behaviors show that a strong retention with SiO_2_ and RP-18 surfaces is a result of the interaction of central ion of analytes with silanol groups.

Results obtained for all newly synthesized heteronuclear complexes on RP-2 plates developed with methanol: water solution was promising. In the stationary phase on surface, there exist fewer residual silanol groups (degree of surface bonded decreased with increase the chain length or organic modifier because of steric effects). The first stage of our investigation was the selection of a chromatographic system, in which retention of chromatographed substances was in the analytical range. For a novel Schiff base complexes, it was achieved on RP-2 HPTLC plates using methanol: water 9:1 (v/v) mobile phase. The results obtained by RP-2 HPTLC gave the possibilities for further investigations. The solvent system was used later for measurement of the partition coefficient between aqueous and stationary phases in reversed-phase liquid chromatography, which is strictly correlated with lipophilicity of a investigated solute.

### Lipophilicity

Schiff base ligands containing various donor atoms (like N, O etc.) show broad biological activities and are of special interest due to variety of ways in which they can bond to metal ions. It is known that the existence of metal ions bonded to biologically active compounds may enhance their activities [5, 30]. In the literature, we can find few works about antitumor, antimicrobial and antiviral activities of some lanthanide binary and ternary complexes and their ligands.

**Fig. 3:**
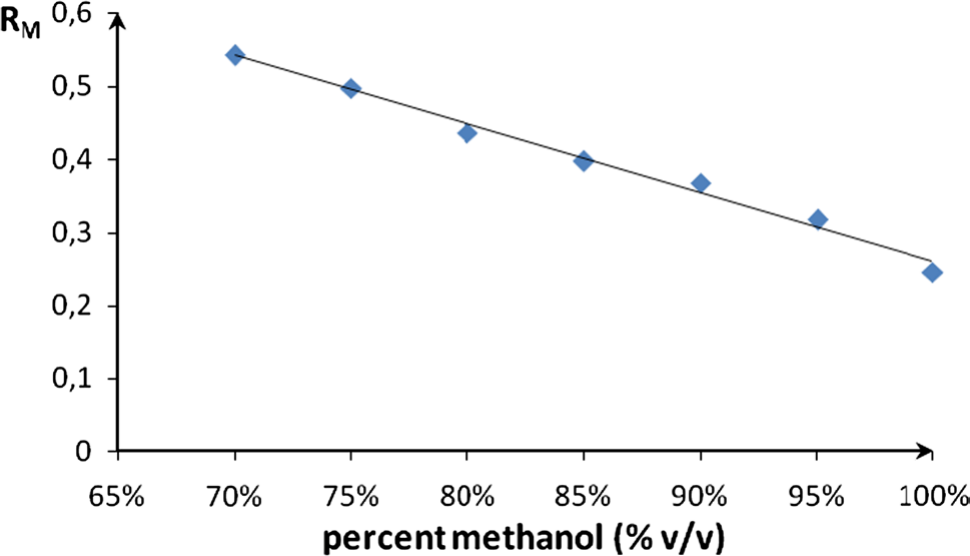
The dependence between *R*_M_ values and concentration of methanol in mobile phase for compound Cu–Eu–Cu

Lipophilicity is one of the most important physicochemical properties of the bioactive molecule. This molecular property affects the penetration of cell membranes, blood–brain barrier distribution, drug adsorption, toxicity, etc. [31]. The relative lipophilicity of 12 complex compounds with Schiff bases as a main ligand was determined by RP-2 HPTLC with mixtures of methanol and water as mobile phase. The *R*_M_ values of the compounds decreased linearly with increasing concentration of methanol in the mobile phase. In order to quantify lipophilicity, the commonly accepted retention parameter used in RP LC systems is log*k*_w_ in column or *R*_M0_ in thin layer chromatography. The parameter was calculated from Soczewinski–Wachtmeister the equation [32]:

2 $$ R_{\text{M}} = \, R_{{{\text{M}}0}} {-} \, S\varphi $$

where *φ* is the volume fraction of organic modifier in the mobile phase, *R*_M_ and *R*_M0_ are retention parameters corresponding to mixed effluent or water as a mobile phase, respectively, and *S* the slope of regression curve. The regression slope *S* is regarded as a characteristic of the specific hydrophobic area of the solute. The influence of *R*_M_ values versus the concentration of organic modifier in mobile phase for compound Cu–Eu–Cu is presented in Fig. 3.

The relative lipophilicity, expressed as *R*_M0_ values and statistical parameters are listed in Table 1. The high value of regression coefficients (*r* > 0.8749) for all 13 compounds proved to be an excellent fit between experimental data and Soczewinski–Wachtmeister equation [33].

**Table 1:** Data for linear correlation between *R*_M_ values and the concentration of methanol [c(% v/v)] in the mobile phase for Schiff base heteronuclear compounds 1–12 and their ligand, and the correlation coefficient (*r*)

	Compound	*R*_M0_	*r*	*S*
1	Cu–Pr–Cu	1.742 (±0.011)	0.9325	−1.48 (±0.12)
2	Cu–Nd–Cu	1.7254 (±0.022)	0.9533	−1.49 (±0.11)
3	Cu–Sm–Cu	1.7077 (±0.015)	0.9495	−1.47 (±0.16)
4	Cu–Eu–Cu	1.1987 (±0.032)	0.9835	−0.94 (±0.21)
5	Cu–Gd	2.0414 (±0.026)	0.8749	−1.9 (±0.15)
6	Cu–Tb	1.9568 (±0.020)	0.919	−1.87 (±0.11)
7	Cu–Dy	1.8421 (±0.023)	0.9296	−1.84 (±0.17)
8	Cu–Ho	1.607 (±0.024)	0.797	−1.45 (±0.25)
9	Cu–Er	1.7667 (±0.024)	0.9391	−1.58 (±0.13)
10	Cu–Tm	1.5777 (±0.012)	0.9061	−1.32 (±0.11)
11	Cu–Yb	1.8058 (±0.016)	0.9792	−1.6 (±0.08)
12	Cu–Lu	1.9086 (±0.021)	0.9768	−1.61 (±0.18)
13	F–L (ligand)^a^	1.6503 (±0.011)	0.9737	−2.76 (±0.19)

^a^RP-18 HPTLC plates

The lipophilicity of ligand must have been determined using RP-18 plates, because in RP-2 system retention was too weak. The differences in the behaviour of the ligand and the metal complexes may be ascribed to the increased lipophilic nature of the complexes arising due to chelation. It is probably due to faster diffusion of the chelates as a whole through the cell membrane or due to the chelation theory. According to the mentioned theory, chelation reduces the polarity of the central ion from partial sharing of its positive charge with the donor groups; п-electron delocalization in this chelating ring also increases the lipophilic nature of the central atom, favoring permeation through the lipid layer of the membrane [30, 34, 35].

The different lipophilicity of the metal complexes compared with that in the ligand may be caused by the strong interaction between the imine moieties and the metal ions. In some investigations, the biological activity of the Schiff base ligand is related to the imine moiety, which plays a key role in the inhibition of some bacteria [5, 36].

The lipophilicity parameters determined by RP-HPTLC were in the range 1.199–2.041. The lowest relative lipophilicity was obtained for compound four. The highest lipophilicity was measured for compound five. Trinuclear Schiff base derivatives, except for Cu–Eu–Cu, have almost the same value of *R*_M0_. In series of closely related dinuclear compounds, the lipophilicity depended from the element being central ion. It was proved that the values of the *R*_M0_ for the investigated dinuclear compounds increased in the following order of their central ion: Tm < Ho < Er < Yb < Dy < Lu < Tb < Gd. It can be explained as follows. The activity of any compound is a complex combination of steric and pharmacokinetic factors. In that reason other physicochemical factors such as solubility, conductivity or dipole moment that are affected by the presence of metal ions may also be the possible reasons for changing the biological activity of different metal complexes.

### The Effect of pH Buffer

**Fig. 4:**
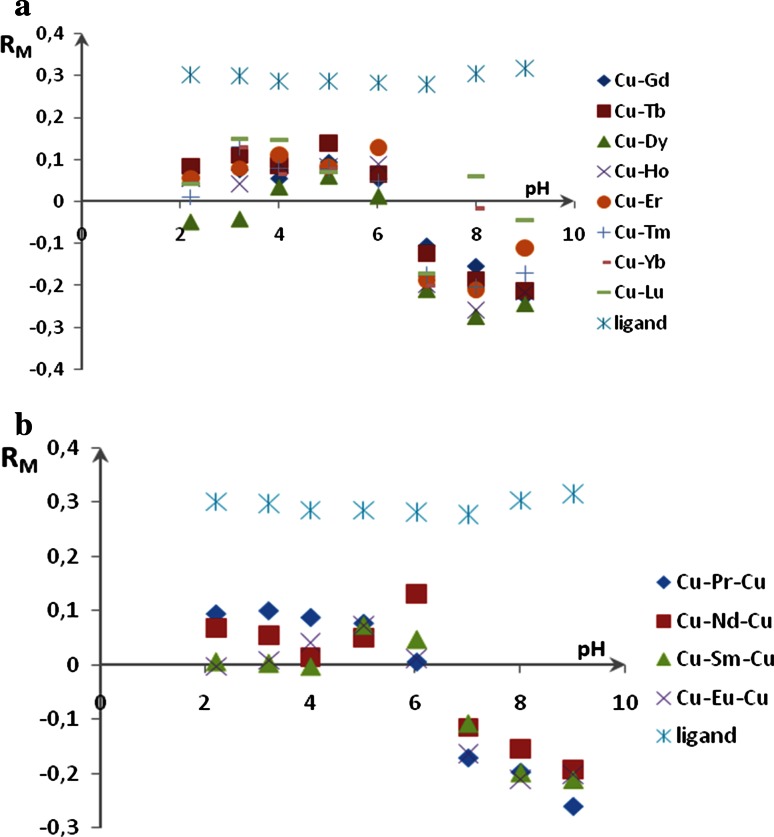
Change of retention with pH for dinuclear (**a**) and trinuclear (**b**) Schiff base derivatives. Mobile phase: methanol: citric buffer 9:1 (v/v)

Mobile phase can have very significant influence on retention and selectivity in reversed-phase chromatography by influencing solute ionization in the mobile phase. The effect of the mobile phase pH on the retention of the novel coordination compounds was investigated in this study by changing the pH of mobile phase solution in the range of 2–10. Figure 4 shows the pH influence on the retention values of all dinuclear (Fig. 4a) and trinuclear (Fig. 4b) Schiff base complexes.

The relationships between retention and mobile phase pH show that retention of all chromatographic complexes remained relatively unchanged in the pH from 2 to 6, but decreases sharply, when the pH increases to 7. All investigated Schiff base derivatives became less hydrophilic which leads to growth of their retention. In contrast to heteronuclear complexes, retention of the ligand was practically the same in the 2–10 pH range.

Research on the influence of mobile phase pH on retention of the newly synthesized Schiff base derivatives proved that the form of investigated compounds in the solution depends on its pH. It means, that when the pH in mobile phase is in the range 2–6 the form of the investigated complexes is different from that in the mobile phase in the pH range 7–10. In order to determine the form of the investigated compounds, further studies were carried out.

### Electrochromatography Measurements

Planar electrochromatography (PEC) is a variant of thin layer chromatography (TLC) where the mobile phase is not only driven by capillary action, but also by electroosmotic flow (EOF). The main advantage of using EOF is that, theoretically, a flat flow profile of the mobile phase is achieved in the whole cross section of sorbent bed (plug-like flow profile). In order to develop the knowledge about the various compounds forms, we studied the retention changes in three different environments:

1. acidic (mobile phase—methanol : buffer pH = 3 9:1 v/v),
2. neutral (methanol: water 9:1 v/v),
3. basic (methanol: buffer pH = 8, 9:1 v/v),

by the use of planar electrochromatography method.

Strong electric field can influence interactions between chromatographic compounds and the stationary or mobile phases. The change retention with change to pH informs us about the changing forms of coordination compounds, but does not give us any information about the sign of ion, while electrochromatographic results enable do it.

Electrostatic field may influence on the retention of substances (including novel synthesized di- and trinuclear coordination compounds) in various ways. Separations using dry layers are performed in a horizontal chamber, two ends of the plate contacts a solvent reservoir which contains an electrode by which an electric field is applied to the plate. Analyzing relations between anode- and cathode-side retention, we may draw the following conclusions:

1. when the retention is stronger in the cathode side than anode, it means that chromatographed substances may be positively charged (cation), because electrophoretic velocity is opposite to the direction on the mobile phase in cathode side and the same in anode side (Fig. 5a);

**Fig. 5:**
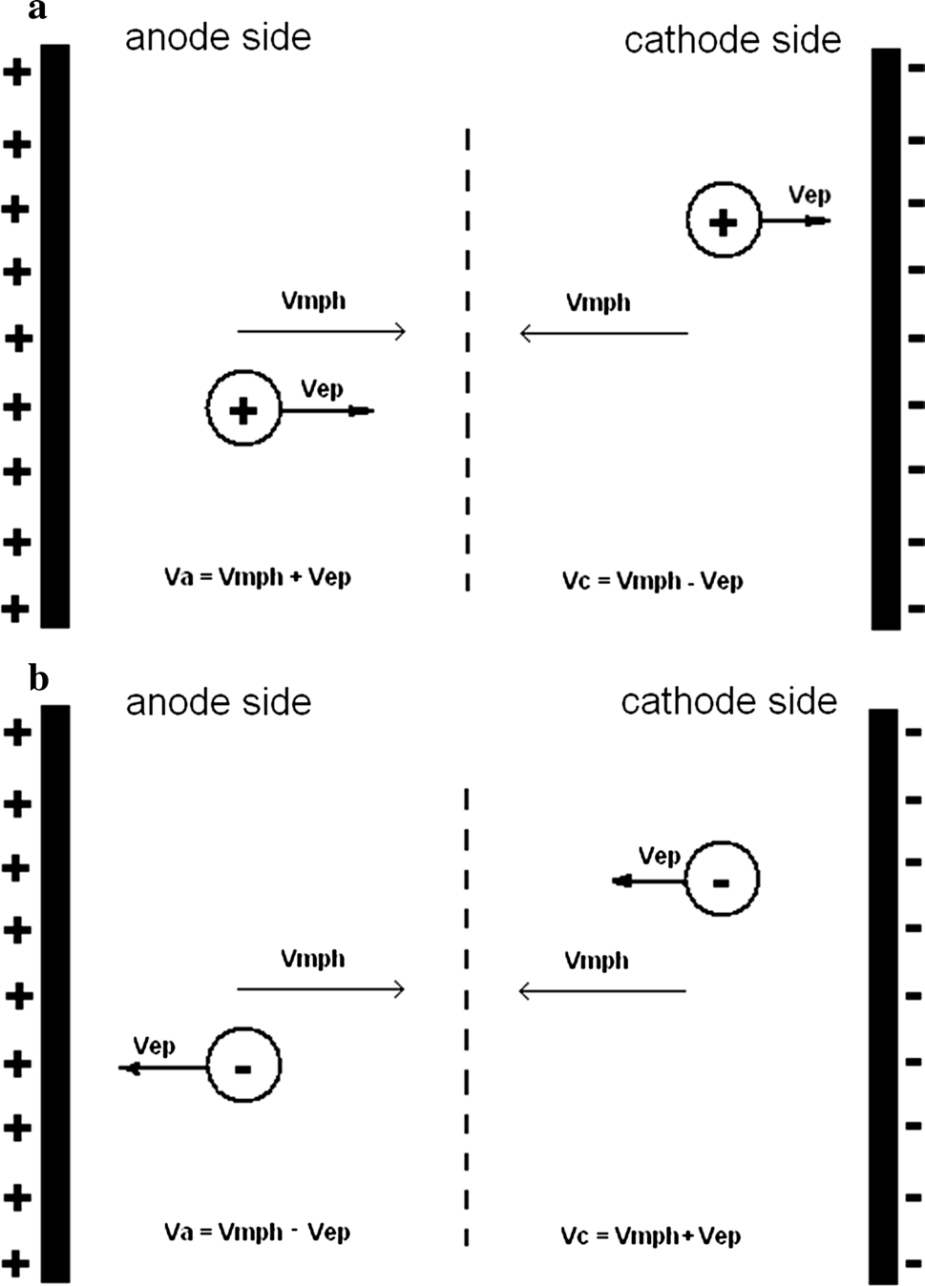
Scheme of the ions migrations in planar electrochromatography process: **a** cation migration, **b** anion migration (*v*_*mph*_ velocity of mobile phase migration, *v*_*ep*_ velocity of electrophoretic migration of ion, *v*_*c*_ cation velocity, *v*_*a*_ anion velocity)
2. when the retention is stronger in the anode side than cathode, it means that chromatographed substances may be negatively charged (anion), since electrophoretic velocity is opposite to the direction on the mobile phase in anode side and the same in cathode side (Fig. 5b);
3. when the retention is practically the same on both sides, it may mean that the substance is neutral.

**Fig. 6:**
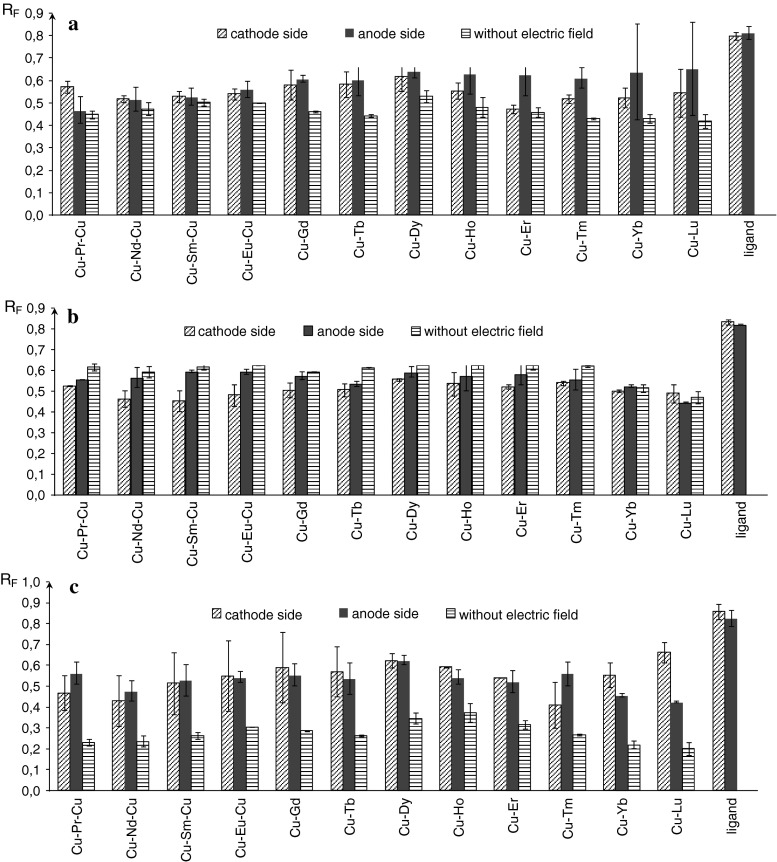
Differences between *R*_F_ values obtained outside and inside electrostatic field for all chromatographic complexes in following mobile phases: **a** methanol: citric buffer pH = 3 9:1 (v/v), **b** methanol: citric buffer pH = 9 9:1 (v/v), **c** methanol: water 9:1 (v/v)

Electrostatic field can also change the retention of neutral substances, because it may change the interaction between all the components of chromatographic system. It has been proved that the retention of chromatographed substances may be different in and out electrostatic field, while the retention in anode and cathode sides is practically the same (Fig. 6a–c).

Under the influence of external electrostatic field in acidic environment [mobile phase methanol: buffer pH = 3 9:1 (v/v)], all investigated heteronuclear complexes migrate faster than without the field (Fig. 6a). Among trinuclear complexes, only Cu–Pr–Cu complex retention is smaller in cathode site compared to anode one, which may suggest that in solution, given complex may have anionic form. Other investigated complexes stay in neutral form. In case of binuclear complexes, substances 8–12, in aqueous solutions have cationic form. Other binuclear complexes in acidic environment remain in neutral form.

Under the influence of external electrostatic field in basic environment [mobile phase methanol: buffer pH = 8 9:1 (v/v)], all investigated heteronuclear complexes migrate slower than in case when the field is absent (Fig. 6b). Retention of compounds: Cu–Nd–Cu, Cu–Sm–Cu and Cu–Eu–Cu are lower in anode side comparing to cathode one, which proves cationic form of these complexes in solution. For dinuclear complexes, the differences between the cathode and anode sides are smaller than standard deviation, thus most probably they are in neutral form.

In Fig. 6c, *R*_F_ values of chromatographed complexes under the influence of external electrostatic field (in cathode and anode side) and without the presence of the field were depicted. A part from earlier described the results where pure water was added instead of buffer [methanol: water 9:1 (v/v)]; *R*_F_ values comprise between 0.4 and 0.7. When electrostatic field is absent, all *R*_F_ values are lower than 0.4. Considering all investigated complexes, the substances with number 1 and 10 migrate significantly higher to anode than cathode side, which implies that they have cationic form in the solution. Substances Cu–Yb and Cu–Lu are stronger retained to anode side compared to cathode one, thus one may say that they are in anionic form in the solution. Ligand retention is very similar on the anode and cathode side, which suggests its neutral form to be confirmed by results of research on mobile phase pH on retention of investigated compounds (Fig. 4). It ought to be mentioned that observed changes are not due to the result of Joule heat generation with absolute certainty, because plate temperature was constantly monitored.

### Magnetochromatography Measurements

Influence of magnetic field on retention of analyzed compounds was investigated using magnetochromatography technique.

Relative ∆*R*_F_ values were calculated using Eq. 3:

3 $$ \varDelta R_{\text{F}} = \frac{{R_{\text{F}}\,({\text{outside magnetic field)}} - R_{\text{F}}\,( {\text{inside magnetic field)}}}}{{R_{\text{F}}\,( {\text{outside magnetic field)}}}} \cdot 100\,\% $$

**Fig. 7:**
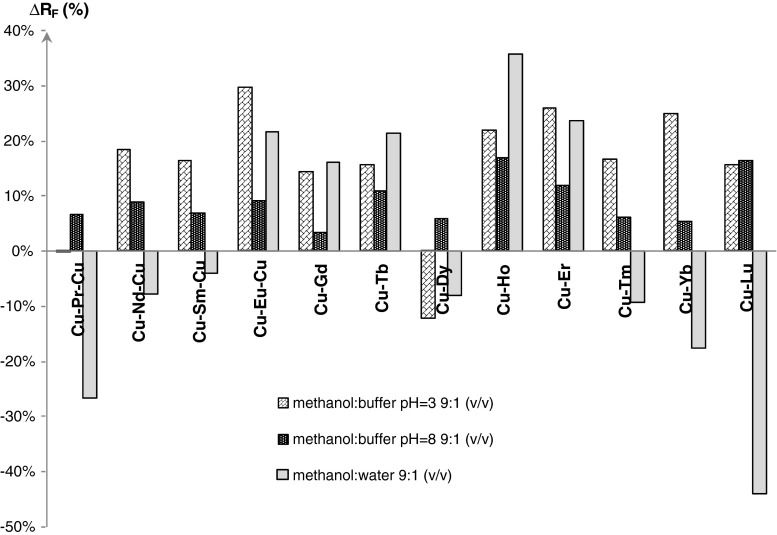
The relative ∆*R*_F_ values obtained for all chromatographic complexes in various mobile phases

Experiment carried out in acidic environment (Fig. 7) proves that retention of majority of the di- and trinuclear complexes is impressed by the factor of field presence. In external magnetic field, where inductivity is about 0.44 T and lines are perpendicular to the mobile phase migration direction, investigated compounds show a more weakly interaction with the stationary phase, and their retention decrease. The only exception is binuclear Cu–Dy complex whose retention is higher in magnetic field than outside of it. Mean retention changes caused by field presence comprise between 15 and 30 %. The most susceptible on magnetic field effect is Cu–Er–Cu trinuclear complex.

In basic environment (Fig. 7), influence of magnetic field on retention is explicit. Applying the magnetic field lowers retention of the compounds in comparison with the retention outside the field, which results from lower interaction between them and stationary phase, but differences do not exceed 18 %. In case of trinuclear complexes, ∆*R*_F_ value is about 6–8 percent while in case of binuclear complexes that strongly depends on the kind of central ion.

The biggest diversity of magnetic field presence effects was observed for methanol: water 9:1 (v/v) mobile phase (Fig. 7). The influence of external magnetic field is totally different for every compound. In trinuclear group of complexes, ∆*R*_F_ values increase in a sequence Cu–Sm–Cu < Cu–Nd–Cu < Cu–Pr–Cu. The only exception is trinuclear Cu–Eu–Cu complex retention of which is higher in magnetic field than outside it, about 20 %. In a dinuclear group of investigated substances, there is also a division for two classes. Firstly, where ∆*R*_F_ > 0, it means that retention in magnetic field is stronger than in system without a external field, Δ*R*_F_ increases in a sequence: Gd < Tb < Er < Ho. Secondly, in which retention in magnetic field is weaker than outside it (Δ*R*_F_ < 0). Sorting the dinuclear compounds (their central ions) by increasing Δ*R*_F_ in external magnetic field, the following list was obtained: Dy < Tm < Yb < Lu.

## Conclusions

In this work, HPTLC, magnetochromatography and electrochromatography were used to examine a group of 12 newly synthesized heteronuclear complexes that include Schiff base and their ligands.

On the basis of the chromatographic measurement and obtained results, it can be concluded that the investigated compounds interact very strongly with silica surface (they practically stayed on the start line in NP system) and have lipophilic properties, because of strong interactions with C-18 and C-8 phase, opposite to ligand. Ionic liquids of the imidazolium tetrafluoroborate added to mobile phase at concentration 1 % (v/v), blocked silanols and decrease retention of all investigation complexes which were otherwise not eluted.

For the determination of lipophilic properties of the investigated compounds, the reversed-phase HPTLC method was optimized and it proved to be applicable and reliable for the analysis of 20 novel complexes based on the *N*,*N*’-bis(5-bromo-3-methoxysalicylidene)propylene-1,3-diamine. The high values of regression coefficients (*r* > 0.8749) proved the sufficient fit between experimental data and the equation: *R*_M0_ = log[(1−*R*_F_)/*R*_F_]. The obtained *R*_M0_ values can be regarded as hydrophobic parameters of the investigated solutes (on RP-2 phase), and their values were found to be in a range of 1.199–2.041.

The studies on the effect of mobile phase pH on retention show that investigated complexes may occur in a few forms in solution. Research on the influence of electric field on retention of the newly synthesized Schiff base complexes was allowed to determine their forms in solution (cation, anion or neutral). Magnetic field can also influence the retention of investigated complexes in RP-2 system. The influence of the external fields (magnetic and electrostatic) depends on mobile phase pH, and in many cases on elements present in central (metal) ions.

Comparing results obtained in conventional RP system and in electrochromatography, it is possible to define the form of the complex appeared in solute and sorbed on the surface of stationary phase can be defined. For example—on the basis of the retention data in conventional RP system it can be concluded, that at pH 2 and 6 the investigated complexes adsorb on the surface as neutral compounds (stronger retention that in the pH range 7–10)—Fig. 4, but electrochromatography measurements in analogical system suggested that some of complexes appear in ion form—for example—complexes Cu–Pr–Cu as anion and dinuclear complexes Cu–Ho, Cu–Er, Cu–Tm, Cu–Yb and Cu–Lu as cation, because significant differences between retention in anode and cathode side are observed (Fig. 6a). It means that, in mobile phase, complexes can form charged associates with buffer solution ions which are destroyed near the surface of stationary phase.

In each of the investigation step, a clear difference can be seen between the properties of ligand and its complexes. The various interactions with the different surfaces are due to the result of blocking the ligand functional group in chelating process.

It has been demonstrated that the chromatographic methods can be used not only as a separation technique, but also as a method for the determination of the physicochemical properties of coordination compounds in solutions, and their affinity to different polar and apolar surfaces.
